# Management of the COVID crisis in Reunion Island (SW Indian Ocean): representations of COVID-19 and acceptance of public health measures

**DOI:** 10.1080/21642850.2023.2252902

**Published:** 2023-09-01

**Authors:** Amandine Junot, Pascale Chabanet, Valéry Ridde

**Affiliations:** a Université de La Réunion, Le Tampon, La Réunion, France; b UMR Entropie (IRD, UR, CNRS, IFREMER, UNC), CS La Réunion, France; c UMR Ceped (IRD, Université de Paris Cité, Inserm), Paris, France

**Keywords:** COVID-19, social representations, health measure, acceptance

## Abstract

**Context:**

The coronavirus pandemic (COVID-19) has caused a major health crisis, requiring the implementation of various public health measures in order to slow the spread of the virus and reduce the associated mortality. However, the success of these measures depends on people’s acceptance of them. This research aimed at understanding people’s representations of COVID-19 and its crisis management, and ultimately at understanding their attitudes toward health measures for counteracting the spread of COVID-19 in Reunion Island together with the behaviours expected of them.

**Method:**

Using Random Digit Dialling, a qualitative study was conducted with 53 inhabitants between February and May 2021. The COREQ checklist was followed. A dual textometric and manual thematic analysis was adopted in order to identify representations of COVID and the management of the crisis.

**Results:**

Some respondents perceived COVID-19 as a serious disease, while others saw it as a banal virus or even doubted its existence. A perceived ineffectiveness of public health measures and the incompetency of public actors predominated in the participants’ discourse.

**Conclusions:**

Thus, there was a considerable lack of trust and negative attitudes toward health measures, possibly influencing people’s acceptance and explaining numerous controversies. This research examines the importance of considering people’s representations of the health situation in order to improve people’s acceptance of protective measures.

## Introduction

Since the first report of Coronavirus Disease 2019 (COVID-19) caused by SARS-CoV-2 virus, the disease spread throughout the world, generating high numbers of infected individuals and deaths in society (∼112.20 million and ∼2.49 million respectively in February 2021) (Kumar et al., 2021). To control the spread, morbidity and mortality of the disease, health authorities and governments have implemented various health measures such as physical distancing, handwashing, mask wearing and vaccination campaigns, or even some more coercive measures such as lockdowns or curfews.

Adherence to public health measures is crucial for successful risk management during pandemics, since it can explain the relative success or failure of health measures deployed by the public authorities. Social determinants, such as socioeconomic status, gender, or age, are crucial factors for predicting health behaviour (Bish & Michie, 2010) and acceptance of health policy. Beyond these socio-demographic criteria, according to cognitive models, people’s acceptance and behaviours are based on an assessment of cost, benefits, risk, usefulness, importance or desirability (Ajzen & Fishbein, 1980; Becker, 1974; Maddux & Rogers, 1983). Indeed, people often have patterns of belief regarding health that determine their health behaviours. The Health Belief Model (HBM) (Becker, 1974; Champion & Skinner, 2008; Ross et al., 2010; Semenza et al., 2011; Wang et al., 2005) was developed to predict the behavioural reaction of individuals in order to predict more general health behaviour. The HBM emphasizes certain beliefs or attitudes toward illness or health behaviours (perceived susceptibility, perceived severity, perceived benefits, perceived barriers, cues to action, and preventive health behaviours) that predict the likelihood of a person adopting the behaviours. This model is a theoretical guideline for health behaviours in public health research. HBM is a good framework for studying health behaviours during the COVID pandemic, with numerous studies confirming the strength of this model to explain why people participate or not in the COVID-19 prevention programme and adopt or not preventive behaviours (Alagili & Bamashmous, 2021; Bavel et al., 2020; Bechard et al., 2021; Shahnazi et al., 2020; Tong et al., 2020; Walrave et al., 2020; Wong et al., 2021; Yehualashet et al., 2021).

According to the HBM, people evaluate how risky a disease is based on its severity and the perceived likelihood of catching it (Bavel et al., 2020; Conner & Norman, 2015; Funk et al., 2009, 2010). The more serious people perceive a disease to be, the more they adopt protective behaviour during a pandemic (Betsch et al., 2015; Bish & Michie, 2010). People also evaluate the risk of health measures, and the perception of the degree of risk is likely to be associated with the adoption of protective behaviours. Beyond risk, people also assess the benefits of measures and their usefulness (Akther & Nur, 2022; Brewer & Fazekas, 2007; Islam et al., 2021; MacDonald, 2015). How health measures can reduce the likelihood or severity of disease, and risk of infection, or how they can even be a solution for preventing pandemics, is important and may influence people’s adherence to measures. Besides benefits, people evaluate convenience of the measures, and perceived inconvenience may lead to difficulties and lower compliance or acceptance.

The COVID crisis has also given rise to conspiracy beliefs, together with distrust of government officials, scientists, and healthcare professionals (Butter et al., 2022; Murphy et al., 2021; Nazlı et al., 2022), all of which may deter people from adherence to public health measures. In decision-making during health crises, organizations and public actors are often judged on their emergency response and reaction to these crises (Faulkner, 2001). Indeed, the extent to which people believe that institutions are competent, able to manage the situation and act responsibly may influence public attitudes toward the health system and protective behaviours, and impact support for the authorities (Freimuth et al., 2014; Gesser-Edelsburg et al., 2020). Among other factors, people’s representations of public authorities can influence trust and ultimately adherence to health measures affecting the population (Bish & Michie, 2010; Freimuth et al., 2014; Muto et al., 2020; Seale et al., 2020).

The objective of this study was to explore the representations of COVID-19 and public health measures in Reunion Island, a French overseas department in the Indian Ocean. Moreover, the aim was to determine the factors which influence if and how the general public adopts recommended behaviours, and to determine their level of confidence in the public authorities.

## Material and methods

### Study site

Reunion Island is a tropical French overseas department located in the southwest Indian Ocean, east of Madagascar. The island had some 905,120 inhabitants in 2022. The Reunionese population is relatively young but is strongly impacted by obesity (11%) and diabetes (9.8%), and is also exposed to a co-epidemic of COVID-19 and dengue (Joubert et al., 2021; Verduyn et al., 2020), raising fears of increasing the number of severe COVID-19 infections (Bruneau et al., 2003). The multicultural population is composed of a co-mingling of several ethnic groups originating from Europe (France, Portugal, and Great Britain), Africa, Madagascar, and Asia (China and India). These immigrations have created a unique lifestyle. Indeed, because of geographic distance and their history, people have developed a strong solidarity network and social capital, i.e. the sense of belonging to a community, which plays a significant role in keeping people on the island (Junot & Praene, 2021; Petrosillo et al., 2013). This vigorous social life could mean a higher risk for transmitting and contracting COVID-19. Finally, the island has experienced a number of epidemics such as malaria (eliminated in 1979), dengue or chikungunya, tropical diseases transmitted by mosquitoes. Thus, the inhabitants and authorities are aware of and prepared for the dangers of emerging epidemic diseases (Taglioni et al., 2013). Yet since the 2005–2006 epidemic of chikungunya, an infectious disease due to an alpha virus transmitted by the tiger mosquito *Aedes albopictus*, and the unexpectedly high mortality from it, there has been a major crisis of confidence in the public health authorities (Boisson et al., 2012)

On March 2020, the island confirmed its first imported COVID-19 case; several clusters of local transmission followed and two years after, 181,000 people have been infected and 540 have died (regional public health authority statistics). As did mainland France and the other French overseas departments, the local authorities deployed health security measures (lockdown, curfews, limitation of movement, restricting or prohibiting people from gathering in groups, hygiene measures such as the wearing of masks in public spaces, etc.). However, non-compliance and protests against restrictive measures have been widespread, such as demonstrations against wearing masks in public spaces and schools.

### Participants

We set a maximum of 60 participants. Sixty participants were merely an estimation of the sample size. This number was thought to be small enough to enable the collection of detailed qualitative data and yet large enough to generate results that could cover a variety of participants regarding quota criteria and also produce maximum depth of data (Kuzel [1992] cited in Saunders, 2012; Cresswell, 2007; Morse, 1994; Sandelowski, 1995). Representative quota samples of Reunion Island were used, stratified by age, gender, and geographical location (municipalities). Consenting participants aged 18 years or older were enrolled. We planned to cease interviews once data empiric saturation had been achieved.

Participants were recruited through a telephone platform between February 2021 and May 2021. For the selection of telephone numbers, we used the Random Digit Dialling (RDD) method. A computer programme randomly generated a list of telephone numbers (*n* = 5000) following the national numbering plan. A second computer programme sent a text message to the list to provide information about the research and notify subscribers that they may be called. We inserted the list into a Reactive Auto Dialler (RAD) to trigger calls in an automatic and optimized way. An audio greeting in French was played to the recipient of the phone call, and then the person was transferred to an employee based in Reunion Island, who explained the research and asked if the person was willing to participate in a semi-structured telephone interview. The participants interested in doing so left their phone number and selected a free slot in the calendar when they would be contacted by the researcher.

### Data collection

Data was collected from semi-structured interviews conducted over the phone. Participants’ representations and attitudes were explored using a semi-structured questionnaire that had been pre-tested with non-participating residents (Appendix 1). The interview began with a summary of socio-demographic criteria (gender, age groups, municipality, and profession). Thereafter, more exploratory questions were asked to address the interviewees’ social perceptions of the health measures and the management of the crisis; to this end, participants were interviewed about their feelings, and how they perceived the advantages and drawbacks of the measures. General open-ended questions were asked, such as ‘what does COVID-19 mean to you?’, ‘what do health measures mean to you?’, ‘what is your opinion of the public authorities and their crisis management?’, ‘which health measures impact your daily life?’ Then, depending on the context of the responses, the interviewer continued with exploratory questions: ‘could you please give an example?’ or ‘could you explain more?’ These questions allowed us to collect respondents’ representations of certain aspects of COVID-19 and measures taken by the authorities (the interview guide can be found in Additional file 1). The Health Belief Model (HBM) was used as a framework for developing questions for the interviews and thus defined attitudes, beliefs, behaviours related to COVID-19, and health measures.

The interviews took about 30 min to complete. All interviews were audio-recorded and professionally transcribed. To maintain confidentiality, participants were assigned a pseudonym and all contextual identifiers were removed from the transcripts.^1^ Moreover, transcripts were not returned to participants for comments because of the anonymization of personal data. We did not have the participants’ names and the phone numbers were deleted after data collection for ethical reasons.

This study followed all the principles of the Declaration of Helsinki on Ethical Principles for Research Involving Human Subjects and all the ethical instructions.

### Rigour and quality criteria

The checklist Consolidated Criteria for Reporting Qualitative Studies (COREQ) was used to execute and evaluate the study (Tong et al. 2007). The first author conducted the phone interviews with 53 sampled attendees. Data were transcribed verbatim and analysed using content analysis and textometric approach. Appendix 2 summarizes the 32 COREQ items in the study.

### Design and data analysis

In order to identify the representations of COVID and crisis management, we conducted textometric analysis with the open-source software IRAMUTEQ, version 07 alpha 2 (acronym of Interface de R for the Multidimensional Analysis of Texts and Questionnaires [Ratinaud, 2009; Ratinaud et al., 2012]) and manual thematic qualitative analysis. We used two complementary methods to compare and contrast the results. This process takes advantage of both the qualitative perspective, anchored in narrative analysis, and the quantitative perspective in texts, assuming that both the number of repetitions and the specific way in which something has been said may play an important role in the text analysis.

Textometric analysis based on classic textual statistics was carried out with IraMuTeq software. Specifically, for this study, the Descending Hierarchical Classification (CHD) method, adopted by Reinert (1990), allowed identification of the words and text segments with the highest Chi square values, that is, the words and text segments that best identify each ‘class’ or idea that the participants have repeatedly mentioned about representations of COVID-19 and management of the health situation. Compared to traditional qualitative analysis, beyond identifying the major themes, CHD brings to the fore the organizational structure of the significant elements of every representation. It also shows the thematic classes that emerged and confirmed connections between them, thus improving the quality of the presentation of outcomes. We analysed the typical vocabulary and extracts for each class, and returned to the text to label and interpret them. The Descending Hierarchical Classification analysis (CHD) provided the structure of the respondents’ representations. The manual thematic qualitative analysis added content to the structure of the mental representation highlighted by the automatic analysis.

The results of the automatic and manual analyses were interpreted conjointly. We performed a manual content analysis with inductive open coding process as developed by Strauss & Corbin (1990). The interviews were read and re-read to identify interesting features of the data and potential themes and compared the identified theme with the themes emerging from the previous software analysis, which allowed the data to be harmonized to one final coding (Appendix 3). This process also furthered understanding of the identified themes and the identification of illustrative quotations. The relevant quotations were reviewed, and the final themes were generated. Moreover, content analysis allowed the highlighting of the arguments used by interviewees to justify their attitudes and behaviours.

In addition, depending on sociodemographic variables, people’s representations can vary. On IRAMUTEQ, Chi-square tests allows difference in class to be observed according to the latent variables, and thus highlighting differences of representations with *χ*^2^ which calculates the strength of association between modalities of latent variables and the identified class. Thus, we analysed the strength of association between modalities of latent variables (age, gender and place of residence) and the identified class.

## Ethics statement

All procedures performed in studies involving human participants were in accordance with the ethical standards of the institutional and/or national research committee and with the 1964 Helsinki Declaration and its later amendments or comparable ethical standards.

Informed consent was obtained from all individual participants involved in the study.

Moreover, the study ‘Management of Covid crisis in Reunion Island’ based a non-interventional study in nature. Thus, it is not subject to the Jardé Law which defines the category of Research Involving Human Participants (RIHP) as ‘research organized and conducted on healthy or sick volunteers, aimed at the development of biological or medical knowledge, and which aims to evaluate: the mechanisms of functioning of the human organism, normal or pathological, the effectiveness and safety of carrying out acts or the use or administration of products for the purpose of diagnosing, treating, or preventing pathological conditions’.

Therefore, we confirm that at a stage of a survey like the one carried out in the study ‘Management of Covid crisis in Reunion island’, there was no legal obligation to submit the project to the opinion of an ethics committee.

In addition, the IRD Ethics Committee is consulted on a voluntary basis and is only advisory.

## Results

### Descriptive statistics

Between February 2021 and May 2021, a total of 53 people agreed to be interviewed and interviews were conducted in parallel. Although 53 people were interviewed out of the 60 participants targeted, we had reached the empiric saturation point at 40 participants.

The sample was composed of 23 men and 30 women, aged between 18 and 78 years old (*M* = 41.2, *SD* = 14.55) (Table 1).

**Table 1.** Characteristics of the sample.

	Saint-André	Saint-Denis	Sainte-Marie	Tampon	Possession	Saint-Pierre	Saint-Joseph	Petite-Ile	Sainte-Suzanne	Saint-Paul	Saint-Louis	Saint-Benoit	Le Port	Plaine des Palmistes	Etang Salé	Total
Average age	33	48.4	42	38.5	33	43.4	45	54	53.5	45.6	43.3	44.7	32.5	37	45	
Men	2	4	1	3	1	3	1	0	1	3	1	1	1	0	1	23
Women	2	5	1	3	1	4	2	1	1	4	2	2	1	1	0	30
Total	4	9	2	6	2	7	3	1	2	7	3	3	2	1	1	53

We analysed representations of COVID-19, health measures and the actors of public health management respectively.

### Representations of COVID-19

**Figure 1.**
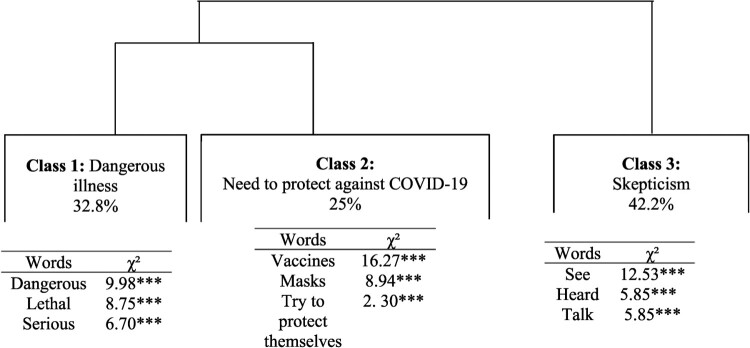
Hierarchical clustering dendrogram of representations of COVID-19.

Through CHD, three classes were identified. Figure 1 shows a dendrogram, in which the active forms contained in the TS associated with each class are indicated. Three major themes emerged from the three classes identified. The first main cluster was formed of Class 1, with a weight of 32.8% (‘a dangerous disease’) and Class 2, with a weight of 25% (‘need to protect against Covid-19’). The second main cluster, with the greatest representation in the corpus, (42.2%), was composed of Class 3 (trivialization and scepticism). Thus, the analysis highlighted two opposing representations of COVID-19. On the one hand, within Classes 1 and 2 people used words such as ‘dangerous disease’, ‘serious illness’, ‘a disease that kills’ or ‘death’, ‘vaccine’, ‘mask’ and ‘be careful’. COVID-19 was perceived as a serious or deadly illness against which you need to be protected: ‘it is a very serious disease, it is a deadly virus’ (woman, 43, Saint-Denis); ‘it is a disease which can be lethal … we try to be careful’ (man, 31, Le Tampon). On the other hand, within Class 3, words such as ‘real’ and expressions such as ‘really exist’ or ‘I don’t see sick people’, revealed people’s scepticism. Some participants doubted the existence of the virus:

apparently it’s a disease, apparently there are numerous cases but I don’t see anything! We’ve heard about cases but we don’t see sick people so we don’t know if it is true or not. I’m wondering if it really exists! (man, 59, Le Tampon)

This doubt was associated with a trivialization of COVID-19 or distrust towards government and media. In fact, the terms ‘simple’ and ‘flu’ cropped up regularly in the discourse, where COVID-19 was compared to a simple dose of the flu: ‘it is an illness like any other illness’ (woman, 31, Saint-Paul); ‘Covid is a trivial virus, it is like the flu’ (woman, 30, Le Tampon). These participants thus perceived COVID-19 as a non-serious illness. Moreover, interviewees sometimes questioned the role of the government and media; according to certain respondents, these actors exaggerated the health situation: ‘I think there’s too much hype’ (woman, 40, La Possession), ‘We live in fear of what we have heard about Covid rather than Covid itself’ (woman, 37, La Plaine des Palmistes), and other people consider it to be a government’s invention: ‘I think that heads of state are the cause of this problem’ (man, 50, Saint-Pierre).

Manual analysis emphasized another theme: that of the constraints imposed to manage the virus: ‘There are many constraints, it’s inconvenient, life is not the same’ (woman, 40, Saint-Joseph); ‘it is a global virus which puts the brakes on the world and which has changed our habits’ (man, 35, Sainte-Marie).

Finally, the gender and age of the respondents did not discriminate the discourse (absence of Chi^2^). Representations of COVID-19 did not vary by gender and age of the individual.

### Representations of the measures

**Figure 2.**
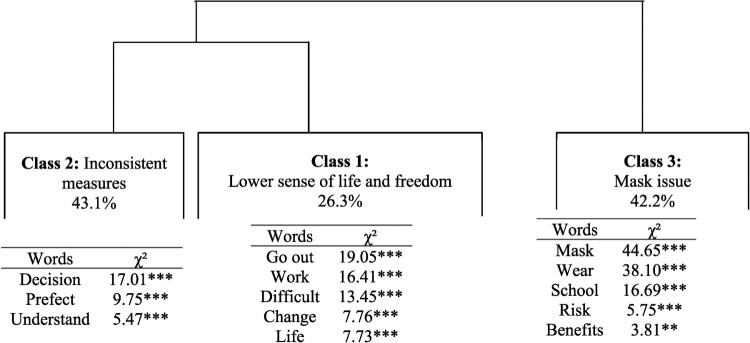
Hierarchical clustering dendrogram of representations of health measures.

Through CHD, three classes were identified. Figure 2 shows a dendrogram, in which the active forms contained in the TS associated with each class were indicated. Three major themes emerged from the three classes identified. The first main cluster was composed of Class 1 with a weight of 26.3% (Lower sense of life and freedom) and Class 2 with a weight of 43.1% (inconsistency of measures). The second main cluster was composed of Class 3 with a weight of 30.7% (the issue of mask wearing).

Within Class 1, people used words such as ‘go out’ (not possible to go out), ‘work’, ‘difficult’, or ‘restriction’. The curfew was perceived as a daily constraint: people felt that they were reduced to working only, and they had no leisure activities. Health measures, especially the curfew, were perceived as difficult because of restrictions in daily life: ‘with the curfew we don’t really live, we work and sleep, 6 pm is too early’ (man, 31, Le Tampon); ‘the curfew is binding, so in relation to extracurricular activities, there is not much for children to do’ (woman, 30, Saint-Louis). Moreover, in the interviews, the terms ‘change’ and ‘life’ appeared. The respondents emphasized a change in habits. The manual thematic analysis showed that the interviewees seemed to be affected in their social and family life, an important aspect of traditional life in Reunion Island: ‘These measures are difficult for me. It’s the end of Sundays, no more family outings and picnics’, (woman, 42, Saint-Pierre); ‘the curfew is disabling in relation to transports and for seeing my family’ (man, 18, Saint-Denis). Class 2 was composed of words such as ‘took’, ‘prefect’, ‘decisions’, or ‘understand’. The manual thematic analysis revealed a misunderstanding of measures due to inconsistency: ‘a curfew at 6 pm is useless because all day long people roam freely and at 6 pm people are already home’ (man, 27, Saint-Pierre), ‘a curfew is not helpful if restaurants and bars are open’ (man, 49, Saint-Paul). Respondents assessed the effectiveness of the curfew by considering exposure of people during the day at work, at school or in the supermarket. Interviewees in these classes perceived a high probability of contamination at school or difficulties in the workplace and thus the impossibility of enforcing barrier gestures. Management of the airport was also pointed to as inconsistent, as the airport was considered by respondents in these classes as the gateway of the virus: ‘a year ago, there were no cases, the airport was closed and now it is not the case and we seem to be losing control of the health situation’ (woman, 31, Saint-Paul).

Finally, Class 3 was composed of terms such as ‘mask’, ‘wearing’, ‘school’, ‘risk’ and ‘benefits’. People in this class criticized compulsory wearing of face masks at school and in daily life. Mandatory masking at school was perceived as difficult or even dangerous, and thus unacceptable: ‘What bothers me the most is compulsory masking at school. I have two children and they have to wear a mask all day long, it’s not easy’ (woman, 30, Saint-Louis); ‘Scientists say that masks could be dangerous for kids because they need more oxygen to grow healthily. Some say that masks could have neurologic consequences’ (woman, 43, Saint-Denis). Mandatory masking at school, in outdoor spaces and work was criticized: ‘wearing a mask is pure bullshit. A surgical mask allows more than 80% of the virus to pass through: it doesn’t prevent infection’ (man, 28, Saint-André).

Gender discriminated the discourse, with Class 3 overrepresented by women between the ages of 25 and 49 (Chi^2^ = 11.15, *p* < .001). Age did not discriminate discourse on the issue of masks and inconsistent measures; however, it discriminated the discourse on daily restrictions. People aged between 42 and 62 emphasized daily restrictions more (Chi^2^ = 5.28, *p* < .05). Moreover, there was no discrimination according to the municipalities of residence (absence of Chi^2^).

### Representations of actors of public health management

**Figure 3.**
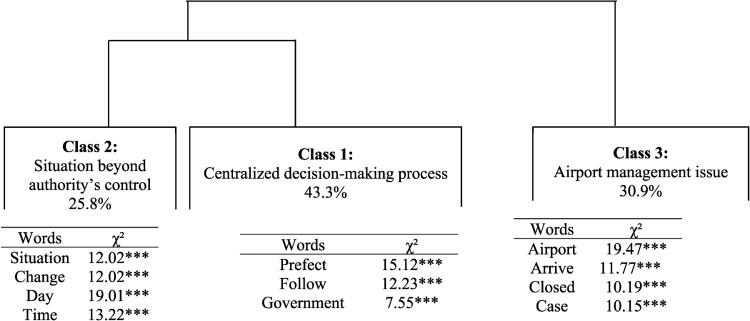
Hierarchical clustering dendrogram of representations of COVID-19 crisis management.

Through CHD, three classes were identified. Figure 3 shows a dendrogram in which the active forms contained in the TS associated with each class were indicated. Three major themes emerged from the three classes identified. The first main cluster was composed of Class 1 with a weight of 43.3% (centralized decision-making process) and Class 2 with a weight of 25.8% (situation beyond the control of the authorities). The second main cluster was composed of Class 3 (airport management issue).

Within Class 1, the discourse contained words such as ‘Prefect’, ‘follow’, and ‘government’. According to certain respondents, management of the COVID crisis was centralized at government level and the Prefect only applied government decisions, as illustrated by the following segments: ‘I think that the Prefect^2^ is obliged to follow government instructions; he is not the one in charge of management’ (man, 55, Saint-Denis); ‘the Overseas Minister has decided, and the Prefect is only there to implement the decisions’ (man, 35, Sainte-Marie). Within Class 2, we noted the words ‘situation’, ‘change’, ‘day’, ‘time’ or ‘measure’: ‘They don’t really know how to manage and the Prefect does not implement the measures; every week he postpones his speech and in the end nothing changes!’ (woman, 20, Saint-Paul). Respondents felt there was a lack of rigour in decision-making, with day-to-day decision-making, or belated decisions: ‘But there are inconsistencies, such as the reaction time and the justification for this reaction!’ (man, 27, Saint-Pierre); ‘They shouldn’t wait more for more cases!’ (man, 37, Saint-Pierre). Ultimately, respondents in Class 2 perceived public actors as overwhelmed by the situation: ‘I think the government is overwhelmed by the epidemic … ’ (woman, 25, Saint-Denis). Finally, in Class 3, words such as ‘airport’, ‘closed’, ‘arrive’, ‘case’ or ‘beginning’ emphasized the airport management issue. The interviewees in this class considered crisis management as bad or even chaotic: ‘The management is chaotic, no airport closure, no lockdown, whereas last year we were in lockdown for less than that’ (woman, 45, Saint-Joseph). The airport was considered as a doorway of the virus and the authorities as responsible for the health situation in Reunion Island.

Age, gender and municipality of residence did not discriminate the discourse (absence of chi^2^).

## Discussion

Our study looked at the representations of COVID-19 and management of the health crisis in Reunion Island. The results of our study confirmed the role of the components of the HBM in health behaviour in the face of COVID-19 and emphasized the possible reasons for rejecting health measures.

The results of this present research supported the role of perceived risk as an important determinant to consider; low perception of risk could be a strong reason for high risk behaviours or non-compliance with health measures. The study brings new understanding of the low perception of risk which is influenced by a trivialization of COVID-19 or even the non-recognition of its existence. Indeed, a majority of participants trivialized COVID-19 and were sceptical. Maybe this trivialization is linked to the fact that in 2020 Reunion Island was less exposed to COVID than the French mainland and ultimately that might have had an influence on the level of respect for lockdown measures and attitudes about being contaminated (Laconi et al., 2021). Also, the literature (Barr et al., 2008; Conner & Norman, 2015; Lau et al., 2007; Leppin & Aro, 2009; Poletti et al., 2012; Qian & Li, 2020; Wise et al., 2020) suggests that perceptions of the safety of the situation could explain opposed behaviours and protest movements on the island. Thus, trivialization is an important aspect to take into consideration by authorities since it can make people impervious to the pandemic, in that they become unaware that the disease can affect them and they can transmit it, which has serious consequences for public health.

According to the HBM model, people evaluate the benefits of and barriers to adopting health behaviours. The results of this present research confirmed this. Consistent with past research on acceptance, the perceived risk of masks and their effectiveness were important concerns during the COVID crisis (Fishbein & Cappella, 2006); mask-wearing behaviours were often negatively perceived. To encourage people to wear masks, it would be necessary to demonstrate their safety and effectiveness. Scientists have to develop more research on masks’ effects on health and their capacity to protect people. Moreover, the ineffectiveness of wearing masks could be explained by an international error of interpretation about the mode of circulation of the virus. In fact, at first, the World Health Organisation announced that ‘Covid-19 is airborne and then declared that the virus was transmitted by droplets (e.g. via coughs, sneezes and contaminated objects)’. The fact that the virus was not airborne could have influenced people’s perception of the usefulness of masks (Greenhalgh et al., 2021). Concerning barriers, the binding character of health measures was emphasized. Health measures were related to restrictions, curtailing important aspects of lifestyle such as traditional social life (picnics, Sunday family reunions). COVID-19 measures might affect elements of people’s sense of identity and could thus be detrimental for compliance. This work confirms the suggestion of Laconi et al. (2021), according to which lifestyle plays a role in how measures are accepted. It emphasizes the importance of considering the Reunionese lifestyle and cultural identity as a significant determinant of the acceptance of measures. Furthermore, these results highlight how little these aspects of acceptance were discussed during the COVID crisis even though these elements can be significant determinants of people’s decisions about health behaviours (Unger, 2011). Therefore, further research is needed to identify elements of cultural identity, understand their effects on health-related behaviours and integrate them in HBM.

Finally, these results confirmed the role of trust in beliefs about health and its influence on health behaviours. Indeed, mistrust could lead to even more negative attitudes toward government responses to the crisis (Georgiou et al., 2020; van der Linden et al., 2020) and reduce general compliance with public health measures (Allington et al., 2021; Allington & Dhavan, 2020; Bierwiaczonek et al., 2020; Swami & Barron, 2021). The results provided more understanding of the reasons for mistrust. Firstly, for some people, COVID is not itself dangerous, but the media and the government make COVID dangerous, as they create a fear of it. Statements by the media and government that are perceived as exaggerated might contribute to conspiracy beliefs. As elsewhere around the world, there was a tendency for people to believe in conspiracies about COVID-19. Thus, the results emphasized the importance of moving away from alarmist and stress-inducing statements which do not promote health (Cambon et al., 2021), and of providing more scientifically-based information on the virus, its spread, and the means of managing it, in order to minimize any sense of conspiracy. Moreover, in our study, the loss of confidence was also the result of perceived inconsistency and contradictions. As mentioned above, the contradictory statements of WHO concerning the mode of circulation might have influenced people’s trust in medical authorities Moreover, at the local authority level, the inconsistencies were the result of a conflict between objectives and rules (Sandström et al., 2020), especially the order to close the airport at a time when there were only very few cases of COVID-19 on the island, and then re-opening when the epidemic was spreading more extensively, or a curfew only in the evening and overnight in order to control rising cases. In general, policy inconsistency affects perceptions of policy (van Engen et al., 2019), and specifically during pandemics (Kim & Tandoc, 2021; Rafkin et al., 2021; Siahaan & Komsiah, 2020; Zhang et al., 2021). These inconsistencies could be confusing for some people, disrupting them from complying with recommended health measures and also discrediting the government and its ability to contain the crisis. Therefore, it is important to identify inconsistencies, to understand the reasons why people think there is inconsistency, and justify the choice of measures implemented. Finally, the study’s results revealed negative attitudes toward the local authorities and their crisis management. The authorities were often criticized for acting too slowly and for adopting centralized protective measures which are often perceived as not being adapted to the local context, especially concerning coordination between the number of cases and measures. Previous research has also noted this top-down management as a critical element in eroding public trust (Adamecz-Völgyi & Szabo-Morvai, 2021; Bollyky et al., 2022; Or et al., 2022; Rozenblum, 2021). Furthermore, this top-down management might have reinforced the political distrust evidenced by the 2018 yellow vest (‘gilets jaunes’) protests against governmental policies and also strong distrust of the mainland and public health administration (Hassenteufel, 2020). In fact, within the context of sociocultural frustrations, some of the population of this overseas department experienced a feeling of being unimportant to the mainland, as over the decades centralized decisions have engendered a distrust of the mainland, whether these decisions be colonial (Ridde et al., 2021), political, or about health measures, especially after the chikungunya crisis. Indeed, after belated and inadequate official intervention in response to the chikungunya epidemic (mainland France intervened one year after the virus’s presence had been documented in Reunion Island), a large proportion of people pointed to the central government’s neglect of its overseas citizens as mentionned by Member of Parliament Audifax in 2006. Ultimately, these feelings have led to general public mistrust in the public health administration, health authorities included (Audifax in 2006; Campbell & Knoll, 2020). These results emphasized the importance of working on trust in overseas territories and acknowledging their specificities. Moreover, further research is necessary to help public actors to identify the optimal level (centralized or decentralized) of action (Algan & Cohen, 2021). Beyond the criticisms of centralized management, respondents did not believe the authorities were capable of coping and some people even accused the local authorities of being responsible for the crisis situation on the island, due to perceived mismanagement of the airport. While the effectiveness of measures at airports to reduce the incidence rate of COVID-19 has been questioned elsewhere (Emeto et al., 2021), in our study, the airport issue on this isolated island was linked to a predominant question during the COVID crisis: economic versus health concerns (Ferragina et al., 2021; Ferragina & Helmeid, 2020). Some people accused the government of favouring the economy over the population’s health. These results tally with the literature on the forming of public opinion, which stresses the role played by the impacts of the crisis on both health and the economy (Carrieri et al., 2021; Setiati & Aswar, 2020). According to the degree of the threat for health or the economy, people make different choices: the greater they perceive the health threat from COVID-19 to be, the more likely they are to prefer strict health policies; likewise, the greater they perceive the economic threat to be, the more likely they are to prefer less stringent policies. It is therefore necessary to understand how people perceive risk, so as to design appropriate, effective policy responses. Moreover, these results emphasized how important it is not to adopt communication based on fear, in order to avoid fuelling citizens’ fears.

With the exception of the work of Laconi et al. (2021) about perceived stress and risk, this work is one of the very few to explore people’s representations of COVID-19 and crisis management in Reunion Island. With its qualitative approach, this work allowed us to identify various elements that appear to be involved in the acceptance of health measures. Owing to the presence of significant differences between the lifestyles and perceptions of mainland residents and those of the overseas residents of Reunion, such work is crucial for providing information on how to manage the crisis.

Some limitations of the present study are to be mentioned. We used telephone interviews for data collection to allow participation during the COVID-19 pandemic. Telephone surveys can also be useful to collect data remotely during infectious disease epidemics. However, despite studies suggesting that data quality is comparable between face-to-face and telephone interviews (Leeuw, 2005; Sturges & Hanrahan, 2004), the lack of in-person contact may have limited the establishing of trust between interviewer and participant. Also, social representation analysis is not limited to textual analysis but extends to behaviours or non-verbal communication, which is not possible with our method of telephone interviews. Future research needs to conduct face-to-face interviews in order to explore the entirety of the respondents’ representations. The nature and small sample size of this qualitative study do not allow for the generalizing of results to the pandemic situation and other populations. However, the internal validity of the study is supported by the robustness of our data collection methods and by the saturation of the themes after 40 interviews.

It should also be noted that this work analysed representations of COVID-19 using a qualitative method via textometric and manual analysis. Yet whilst the textometric analysis allowed us to quantify the importance given to the different terms and identified the important classes of words, the manual analysis was a further addition to this analysis of COVID-19 representations, as it enables analysis of the links between the contents, emphasizes opinions, and determines the complexity of language, and thus interprets the meanings of the discourse of each person. This complementary process of qualitative data analysis could be a strong reliable process for analytical credibility.

## Conclusion

This study emphasized the importance of eliciting people’s representations during a health crisis. The different elements of representations of COVID-19 and crisis management could be a useful starting-point for researchers and implementors, who would include them in future models of acceptance of health policy in Reunion Island, and for the development of more successful health approaches in the present or the future. This work emphasized the limits of a policy system which develops measures without considering people’s representations and needs and thus limits people’s acceptance of measures. Understanding patterns of representations can provide tools and guidance for future crises or outbreaks, so that they can be responded to in an adequate manner and so that misunderstandings can be avoided through a participatory approach and appropriate communication.

## Notes

1 Data are available on https://dataverse.ird.fr/dataset.xhtml?persistentId=doi:10.23708/VMWX3N. Data file is provided on request to researchers. A commitment to comply with specific conditions of use is required (purpose, data confidentiality and safety, limited duration …).

2 Senior official representing the government.
